# Neutrophil extracellular traps modulate inflammatory markers and uptake of oxidized LDL by human and murine macrophages

**DOI:** 10.1371/journal.pone.0259894

**Published:** 2021-11-19

**Authors:** Andreas Conforti, Thorsten Wahlers, Adnana Paunel-Görgülü

**Affiliations:** Department of Cardiothoracic Surgery, Heart Center of The University of Cologne, Cologne, Germany; Showa University School of Pharmacy, JAPAN

## Abstract

Neutrophil extracellular traps (NETs) are web-like structures, which are released upon neutrophil activation. It has previously been demonstrated that NETs are present in atherosclerotic lesions of both humans and animal models thus playing a decisive role in atherosclerosis. Besides, macrophages have a crucial role in disease progression, whereby classically activated M1 macrophages sustain inflammation and alternatively activated M2 macrophages display anti-inflammatory effects. Although NETs and macrophages were found to colocalize in atherosclerotic lesions, the impact of NETs on macrophage function is not fully understood. In the present study, we aimed to investigate the effect of NETs on human and murine macrophages in respect to the expression of pro-inflammatory cytokines, matrix metalloproteinases (MMPs) and uptake of oxidized LDL (oxLDL) *in vitro*. Human THP-1 and murine bone marrow-derived macrophages were cultured under M1 (LPS + IFN-γ)- and M2a (IL-4)-polarizing culture conditions and treated with NETs. To mimic intraplaque regions, cells were additionally cultured under hypoxic conditions. NETs significantly increased the expression of *IL-1β*, *TNF-α* and *IL-6* in THP-M1 macrophages under normoxia but suppressed their expression in murine M1 macrophages under hypoxic conditions. Notably, NETs increased the number of oxLDL-positive M1 and M2 human and murine macrophages under normoxia, but did not influence formation of murine foam cells under hypoxia. However, oxLDL uptake did not strongly correlate with the expression of the LDL receptor CD36. Besides, upregulated MMP-9 expression and secretion by macrophages was detected in the presence of NETs. Again, hypoxic culture conditions dampened NETs effects. These results suggest that NETs may favor foam cell formation and plaque vulnerability, but exert opposite effects in respect to the inflammatory response of human and murine M1 macrophages. Moreover, effects of NETs on macrophages’ phenotype are altered under hypoxia.

## Introduction

Atherosclerosis is recognized as the primary pathophysiology of cardiovascular disease and the leading cause of morbidity and mortality worldwide. Lipid-driven vascular chronic inflammation is a hallmark of atherosclerosis and many different cell types, including granulocytes, monocytes/macrophages, dendritic cells, T-cells, B-cells and mast cells contribute to disease progression [1]. The initiating step in the development of atherosclerosis is the accumulation of low-density lipoproteins (LDL) that become sequestrated in the subendothelial space and trigger inflammatory responses inducing monocyte attraction. These monocytes subsequently differentiate into macrophages that scavenge oxidized LDL resulting in the formation of the foam cells. Foam cell buildup contributes to plaque lipid storage and sustained plaque growth [2].

Macrophages are characterized by their high plasticity, which allows them to rapidly response to local microenvironment stimuli. Initially, macrophages were classified as either pro-inflammatory M1 macrophages or alternatively activated M2 subsets, which can be further subdivided into four distinct phenotypes (M2a, M2b; M2c, M2d) [3]. Meanwhile, a wide spectrum of intermediary macrophage phenotypes was identified within the atherosclerotic plaque [4]. Macrophages populating the atherosclerotic plaque have a decreased ability to migrate, which leads to failure of inflammation resolution and to further progression of the lesion into complicated atherosclerotic plaque [5]. They secrete numerous pro‐ but also anti-inflammatory mediators, prothrombotic tissue factor and enzymes such as matrix‐degrading proteases (MMPs), all of which influence plaque growth, cellular composition and stability. Excessive LDL uptake and macrophage apoptosis lead to necrotic core formation in progressive atherosclerotic plaques [6]. In human lesions, M1 macrophages were identified in rupture-prone regions and M2-like macrophages in stable regions and the adventitia [4, 7, 8]. Similarly, macrophage polarization to the M2 phenotype is required for plaque stabilization and regression in mice [9]. Plaque rupture underlies the majority of myocardial infarctions (MIs) and strokes [10]. Ongoing inflammation can limit interstitial collagen production and other ECM molecules and augment the activity of ECM-degrading enzymes like MMPs that can provoke plaque instability, rupture and subsequent thrombus formation [11, 12]. Indeed, increased activity and expression of MMP-7, MMP-9 and MMP-2 was observed in unstable plaques of patient with coronary atherosclerosis [13]. MMP-9 has been identified as a predictor of cardiovascular mortality in patients with coronary artery disease [14]. Besides, MMP-8 expressed by macrophages, among others, was found to be associated with plaque rupture in both mice [15] and humans [16].

Recently, neutrophils have been recognized as important regulators in atherosclerosis and particularly in atherothrombosis [17]. Many stimuli including hyperlipidemia trigger the formation of neutrophil extracellular traps (NETs) by neutrophils located in atherosclerotic lesions [18, 19]. NETs are composed of extracellular web-like decondensed chromatin decorated with various neutrophil-derived proteins. Megens *et al*. first reported the presence of NETs in mouse and human atherosclerotic lesions [20]. In *ApoE*^*-/-*^ mice, NETs were found to prime macrophages for production of interleukin (IL)-1β and IL-6 resulting in the activation of a T helper cell (T_h_) 17 response, which amplifies immune cell recruitment into atherosclerotic lesions [19]. Moreover, it was recently reported by our group that NETs drive murine macrophages towards an anti-inflammatory M2-like phenotype *in vitro* [21]. Although many studies have been exploring the pathophysiology of NETs, their impact on macrophage phenotype in atherosclerosis remains poorly understood. In the present study, we investigated the effects of NETs on the expression of cytokines and MMPs in human THP-1-derived as well as bone marrow-derived murine macrophages *in vitro*. We further explored the uptake of LDL by these cells upon NETs exposure.

## Material and methods

### THP-1 macrophage differentiation and polarization

Human monocytic THP-1 cells (DSMZ, Germany) were cultured in 2 ml RPMI 1640 medium (PAN Biotech) supplemented with 10% FCS and 100 U/ml penicillin and 10 μg/ml streptomycin (Sigma Aldrich) at a density of 2.5 × 10^5^ cells / ml. For macrophage differentiation, THP-1 cells were treated with 5 ng/ml phorbol 12-myristate 13-acetate (PMA; Sigma Aldrich) for 48 h followed by 96 h of resting in PMA-free RPMI medium. Macrophages were further polarized into M1-like macrophages with 100 ng/ml LPS (Sigma Aldrich) and 20 ng/ml recombinant human IFN-ɣ (Peprotech). Polarization into the M2a-like phenotype was obtained by incubation with 20 ng/ml recombinant human IL-4 (Peprotech) for 24 h. Macrophage differentiation and polarization were evaluated by flow cytometry and Real-time PCR (S1 Fig).

### Isolation, polarization and characterization of murine macrophages

Eight to twelve weeks old C57BL/6J mice were euthanized by cervical dislocation to minimize suffering. Isolation of bone marrow-derived cells and *in vitro* differentiation into macrophages were performed as previously reported [22]. Cell isolation from mice was approved by the local ethics committee (Bezirksregierung Köln; Germany; No: 4.17.026; 4.18.015). For *in vitro* polarization, M0 macrophages were cultured in RPMI 1640 medium with 10% FCS supplemented with 20 ng/ml recombinant murine IFN-ɣ (Peprotech) and 100 ng/ml LPS (Sigma Aldrich) to induce a M1-like phenotype or in medium supplemented with 20 ng/ml recombinant murine IL-4 (Peprotech) to induce M2a-like macrophages, respectively. Macrophage phenotype was routinely confirmed by flow cytometry and Real-time PCR as previously described by our group [22].

### Isolation of human and murine neutrophils

Human neutrophils were isolated by discontinuous density gradient centrifugation on Percoll (GE Healthcare) as previously reported [23]. Isolation of human blood cells was approved by the Ethics Committee of the Medical Faculty of the University of Cologne (#15–393). Study participants provided written informed consent. Murine neutrophils were isolated from the bone marrow of 8–12 weeks old C57BL/6J mice, by flushing cells out of the bone with HBSS (Gibco) + 3% FCS. Erythrocytes were lysed using 0.2% NaCl solution, followed by the addition of 1.2% NaCl to restore isotonicity. Neutrophils were isolated by density gradient centrifugation on Percoll (GE Healthcare) at 1000 × g for 30 min at 15°C without break. The pellet enriched in neutrophils was washed twice with HBSS + 3% FCS.

### Generation of NETs-enriched supernatants

Human or murine neutrophils (2 × 10^6^ / well) were cultured in RPMI 1640 medium supplemented with 2% FCS. For induction of NETosis, cells were incubated with 100 nM PMA (Sigma Aldrich) for 3.5 h. Afterwards, cells were carefully washed with PBS and NETs were collected in PBS by vigorous pipetting. To prepare large heterogeneous DNA/NETs fragments [24], *Alu I* (New England Biolabs) was added to the culture medium for 20 min before NETs harvesting. NETs were stored at -80°C until use. The concentration of NETs was quantified using a NanoDrop stectrophotometer (Thermo Fisher) at a wavelength of 260 nm. NETs-associated proteins were quantified by BCA assay (Thermo Fisher).

### Immunofluorescence staining of NETs

Freshly isolated human or murine bone marrow-derived neutrophils (1.5 × 10^5^) were seeded on polylysine-coated glass coverslips (Ø 18 mm), allowed to settle for 30 min, and then treated with 100 nM PMA. After 3.5 h of activation, cells were fixed with 4% paraformaldehyde (PFA), blocked with 5% normal goat serum (Cell Signaling Technology) and 1% bovine serum albumin (BSA) (Carl Roth) and incubated with polyclonal antibodies against myeloperoxidase (MPO) (Abcam) for 2 h at room temperature. Cells were further incubated with a secondary anti-rabbit Alexa Fluor 488-conjugated antibody (Cell Signaling Technology) for 1 h, counterstained with DAPI and mounted in Dako fluorescent mounting medium (Dako). Cells were examined with an inverted microscope (Eclipse Ti-U 100, Nikon).

### Determination of peroxidase activity

The MPO activity in NETs-enriched supernatants was measured by mixing 50 μl sample with 50 μl detection solution (1:1 TMB Substrate A/TMB substrate B, BioLegend) in a 96-well microplate. After 3 min incubation at room temperature the staining reaction was stopped by adding 50 μl H_2_SO_4_. The absorbance of the reaction product was measured at 450 nm using a microplate reader (Victor X3, Perkin Elmer).

### Cell culture conditions

Human THP-1 macrophages were cultured in 6-well plates in RPMI 1640 medium supplemented with 10% FCS and 100 U/ml penicillin and 10 μg/ml streptomycin under normoxic and hypoxic conditions in the presence or absence of human NETs (1000 ng/ml). Murine macrophages were cultured under the same conditions and in the presence of murine NETs (1000 ng/ml). For hypoxia exposure, culture dishes were placed in a hypoxia chamber (STEMCELL Technologies), pre-flushed with a gas mixture (2% O_2_, 5% CO_2_, and 93%N_2_). Macrophages were placed at 37°C in a 21% O_2_, 5% CO_2_, and 74% N_2_ humidified incubator and cultured under standard culture conditions in parallel.

### Real-time PCR

Total RNA was extracted using RNeasy Mini Kit (Qiagen) according to the manufacturer’s instructions. Contaminating DNA was removed by DNA-*free* Kit DNA Removal Kit (Ambion). RNA was reverse transcribed using High Capacity cDNA Reverse Transkription Kit (Applied Biosystems). Primer sequences used for Real time PCR are listed in Table 1. All samples were run in triplicates. Relative gene expression levels were determined using Power SYBR Green PCR Master Mix (Applied Biosystems) according to the manufacturer’s recommended protocol with following thermal cycling conditions: 2 min 50°C, 2 min 95°C, 40 cycles of 1 sec 95°C and 30 sec 60°C, and 4°C hold (QuantStudio 3 Real-Time PCR System, Applied Biosystems). Expression of target genes was normalized to the endogenous control *18S* RNA gene. Fold expression was calculated using the 2^-ΔΔCT^ method. In some experiments, the 2^ΔCT^ method was used to determine relative gene expression.

**Table 1:** Sequences of primer pairs used for Real-time PCR.

Genes	Primer sequences (forward/reverse)
*Human IL-1β*	5’-GTGGCAATGAGGATGACTTGTTC-3’
	5’-TAGTGGTGGTCGGAGATTCGTA-3’
*Human IL-6*	5’-AGCCACTCACCTCTTCAGAAC-3’
	5’-GCCTCTTTGCTGCTTTCACAC-3’
*Human IL-10* ^*b*^	5’-GTGATGCCCCAAGCTGAGA-3’
	5’-CACGGCCTTGCTCTTGTTTT-3’
*Human IL-12*	5‘-AACTTGCAGCTGAAGCCATT-3‘
	5‘-AGGGTACTCCCAGCTGACCT-3‘
*Human TNF-α*	5‘-CTGCTGCACTTTGGAGTGAT-3‘
	5‘-AGATGATCTGACTGCCTGGG-3‘
*Human MMP-2*	5‘-GCTGGCTGCCTTAGAACCTTTC-3‘
	5‘-GAACCATCACTATGTGGGCTGAGA-3‘
*Human MMP-8*	5‘-CCACTTTCAGAATGTTGAAGGGAAG-3‘
	5‘-TCACGGAGGACAGGTAGAATGGA-3‘
*Human MMP-9*	5‘-GCACGACGTCTTCCAGTACC-3‘
	5‘-GCACTGCAGGATGTCATAGGT-3‘
*Human CD36*	5‘-TCTTTCCTGCAGCCCAATG-3‘
	5‘-AGCCTCTGTTCCAACTGATAGTGA-3‘
*Murine IL-1β*	5‘-AGTTGACGGACCCCAAAAGAT-3‘
	5‘-GTTGATGTGCTGCTGCGAGA-3‘
*Murine IL-6*	5’-CCACTTCACAAGTCGGAGGCTTA-3’
	5’-GCAAGTGCATCATCGTTGTTCATAC-3’
*Murine IL-10*	5‘-AGCCTTATCGGAAATGATCCAGT-3‘
	5‘-GGCCTTGTAGACACCTTGGT-3‘
*Murine IL-12*	5‘-GGTCACACTGGACCAAAGGGACTATG-3‘
	5‘-ATTCTGCTGCCGTGCTTCCAAC-3‘
*Murine TNF-α*	5‘-CCGATGGGTTGTACCTTGTC-3‘
	5‘-GGGCTGGGTAGAGAATGGAT-3‘
*Murine MMP-2*	5‘-AACTACGATGATGACCGGAAGTG-3‘
	5‘-TGGCATGGCCGAACTCA-3‘
*Murine MMP-8*	5‘-GATTCAGAAGAAACGTGGACTCAA-3‘
	5‘-CATCAAGGCACCAGGATCAGT-3‘
*Murine MMP-9*	5‘-TCACCTTCACCCGCGTGTA-3‘
	5‘-GTCCTCCGCGACACCAA-3‘
*Murine CD36*	5‘-GCGACATGATTAATGGCACA-3‘
	5‘-CAATGTCCGAGACTTTTCAACA-3‘
*Human / Murine 18S RNA*	5‘-CGGCTACCACATCCAAGGAA-3‘
	5‘-GCTGGAATTACCGCGGCT-3‘

### ELISA

For quantification of IL-1β and MMP-9 in cell culture supernatants, the following ELISA kits were used: Mouse Total MMP-9 DuoSet ELISA (R&D Systems), LEGEND MAX Human MMP-9 ELISA Kit (BioLegend), ELISA MAX Deluxe Set Human IL-1β (BioLegend), MAX Standard Set Mouse IL-1β (BioLegend). All ELISAs were performed following the manufacturer’s instructions.

### Flow cytometry

For characterization of differentiated and polarized macrophages, dead cells were first excluded by live/dead staining (Ghost Dye Violet 510 Viability Dye, Cell Signaling Technology) and single cells were gated by plotting the height against the area of FSC. Cells were single stained with the following antibodies: PE anti-mouse/human CD11b (M1/70; BioLegend), APC anti-human CD163 (GHI/61; BioLegend), FITC anti-human CD197 (G043H7; BioLegend), FITC anti-human CD206 (15–2; BioLegend), PE anti-human CD36 (5–271, BioLegend), FITC anti-mouse CD86 (GL1; BD), Brilliant Violet 421 anti-mouse CD206 (C068C2, BioLegend). The percentage of positive cells and the median of fluorescence intensity were quantified by flow cytometry using FACS Canto II (BD). A minimum of 30,000 cells was assessed. Data were acquired and analyzed using FACSDiva software (BD).

To measure the incorporation Dil-labeled oxidized LDL (oxLDL) by M1- and M2a-like macrophages, cells (1–2 × 10^5^) were cultured for 24 h in HBSS supplemented with 0.3% bovine serum albumin in the presence or absence of 1000 ng/ml NETs. Next, cells were incubated for 6 h with 20 μg/ml Dil-oxLDL (Thermo Scientific) at 37°C, washed twice with PBS and resuspended in 400 μl PBS + 2% FCS+ 1 mM EDTA for immediate FACS analysis.

### Cellular Dil-oxLDL uptake

THP-1 macrophages and murine macrophages were seeded on coverslips (Ø 18 mm) at a density of 1 × 10^5^ cells / well and polarized into M1- and M2a-like cells. Then, cells were washed with HBSS and incubated in HBSS + 0.3% BSA in the presence or absence of NETs (1000 ng/ml) for 24 h. Subsequently the cells were incubated with Dil-oxLDL (20 μg/ml) in HBSS + 0.3% BSA for 6 h. After washing with PBS, cells were fixed with 4% paraformaldehyde for 10 min, counterstained with DAPI and mounted in Dako fluorescent mounting medium (Dako).

### Statistical analysis

Data are presented as mean ± standard deviation (SD). Statistical analysis was performed using GraphPad Prism software (GraphPad Software, San Diego, CA). Data sets were assessed for normality using the Shapiro-Wilk test. Nonparametric unpaired data of multiple groups were analyse using Kruskal-Wallis test with Dunn’s post-hoc test. Unpaired data from two groups were analysed using *t* test or Mann-Whitney nonparametric test. One-sample *t* test (for data that were normally distributed) or Wilcoxon signed-rank test (for data that were not normally distributed) were used when samples were compared with reference control sample (set as 1). When normality could not be assumed, a nonparametric test was performed. A P-value less that 0.05 was considered as statistically significant. The statistical tests used are indicated in the respective figure legends.

## Results

### NETs modulate cytokine expression in human and murine M1-like macrophages

**Fig 1:**
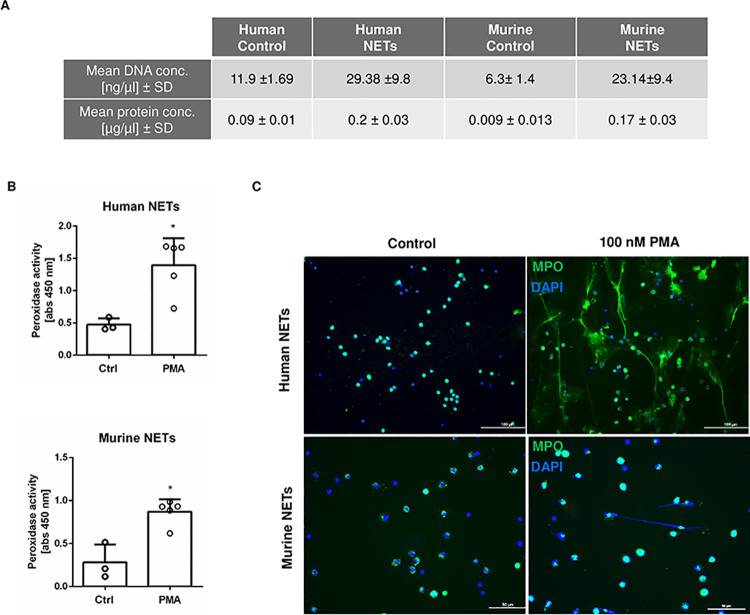
Induction and validation of NETs release by human and murine neutrophils. (A) Human and murine neutrophils (2 × 10^6^) were stimulated with 100 nM PMA for 3.5 h. Then, the DNA and protein contents of NETs-enriched supernatants were quantified (n = 5). Unstimulated cells (n = 3) served as controls. (B) Peroxidase activity was quantified in the supernatant collected from unstimulated (n = 3) or PMA-stimulated neutrophils (n = 5), respectively. (C) To confirm NETs formation, neutrophils (1.5 × 10^5^) were seeded on polylysine-coated coverslips and stimulated with 100 nM PMA for 3.5 h. Cells were further stained with anti-MPO antibody (*green*) and counterstained with DAPI (*blue*). Results of three independent experiments are depicted. *P < 0.05 (Mann-Whitney test).

Human and murine NETs were generated as described in Material and Methods. The formation of NETs was confirmed by DNA, protein and peroxidase activity quantification in NETs-enriched supernatants as well as by immunofluorescence (Fig 1).

THP-1 macrophages and murine macrophages (M0) were polarized by LPS + IFN-γ into the M1 state. Polarization of human THP-1 macrophages was further confirmed by flow cytometry and Real-time PCR, respectively (S1 Fig). THP-M1 macrophages expressed high levels of the M1 marker CD197 and lower levels of the M2 markers CD163 and CD206. Besides, M1-like cells showed increased expression of the pro-inflammatory genes *IL-1β*, *TNF-α*, *IL-6* and *IL-12*. Polarized murine macrophages were previously characterized in detail by our group [22].

**Fig 2:**
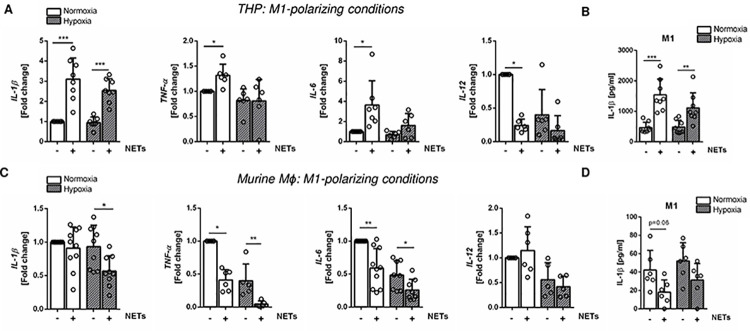
Impact of NETs on the expression of pro-inflammatory cytokines by human and murine macrophages. THP-1-derived macrophages and murine bone marrow-derived macrophages were cultured in the presence of LPS + IFN-γ and human or murine NETs (1000 ng/ml), respectively, for 24 h. In parallel experiments, cells were cultured under hypoxic conditions (2% oxygen). Expression levels of *IL-1β*, *TNF-α*, *IL-6* and *IL-12* as well as IL-1β protein secretion were analyzed by Real-time PCR and Elisa in human THP-M1 (A, B; n = 6–8) and murine M1 (C, D; n = 5–10) macrophages. *P < 0.05, **P < 0.01, ***P < 0.001 (Unpaired *t* test, Mann-Whitney test, One-sample *t* test, Wilcoxon signed-rank test).

To study the capacity of NETs to modulate cytokine expression by macrophages, cells were treated with NETs purified from PMA-stimulated neutrophils in the presence of LPS + IFN-γ under standard culture conditions. As hypoxia is present in atherosclerotic plaques [25], cells were cultured in 2% oxygen in parallel. As depicted in Fig 2, NETs significantly increased the expression of the pro-inflammatory cytokines *IL-1β*, *TNF-α* and *IL-6* in THP-1 macrophages under M1-polarizing conditions and at high oxygen levels. In turn, the expression of *IL-12* was significantly suppressed (Fig 2A). IL-1β levels in culture supernatants also increased significantly (Fig 2B). Notably, the effects of NETs on cytokine gene expression were less pronounced under hypoxic culture conditions and only IL-1β was significantly upregulated. Different results were obtained after stimulation of murine macrophages with NETs. As shown in Fig 2C, NETs strongly suppressed the expression of the pro-inflammatory cytokines *IL-1β*, *TNF-α* and *IL-6* under normoxia and hypoxia, but did not alter *IL-12* expression. In addition, *IL-1β* expression was found to be reduced at 2% oxygen, although no significant downregulation of IL-1β secretion could be observed (Fig 2D). These results suggest that NETs might dampen the inflammatory response in murine macrophages, but conversely increase the pro-inflammatory action of THP-1-derived M1-like cells.

### NETs amplify oxLDL uptake by macrophages

**Fig 3:**
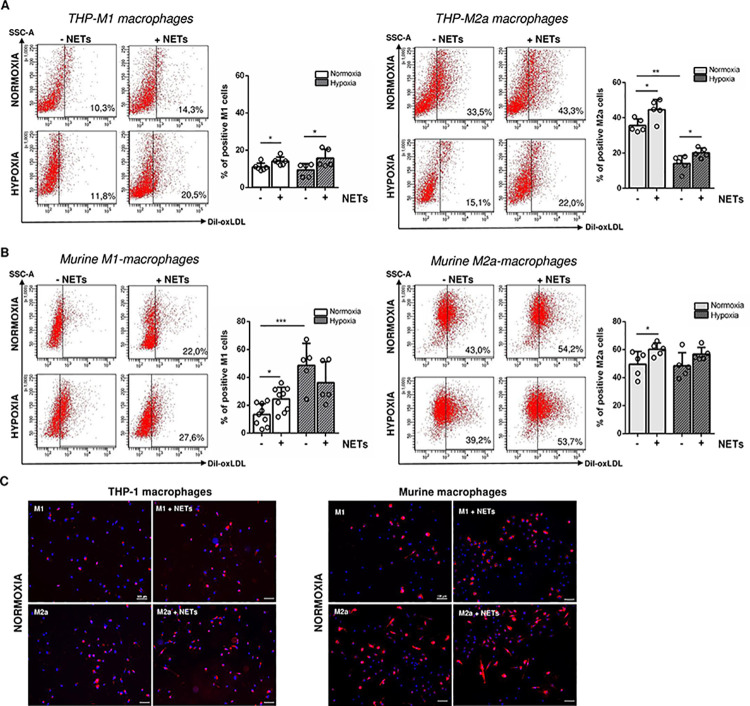
Effect of NETs on oxLDL uptake by polarized human and murine macrophages. The uptake of Dil-labeled oxidized LDL (oxLDL) by human (A) and murine (B), polarized macrophages (M1 and M2a) treated with NETs and cultured under normoxic or hypoxic culture conditions was quantified by flow cytometry. n = 5–9. Representative dot plots are depicted. (C) Dil-oxLDL uptake under normoxia was visualized and confirmed by immunofluorescence. Representative images of three independent experiments are depicted. *P < 0.05, **P < 0.01, ***P < 0.001 (Unpaired *t* test, Mann-Whitney test).

To further explore the effect of NETs on foam cell formation during atherogenesis, polarized THP-1 and primary murine macrophages were incubated in serum-free culture medium in the presence of NETs for 24 h. Although the expression of M1 (CD197, CD86) and M2 (CD197, CD206) markers significantly changed in serum-starved M1 macrophages, it was found to still correlate with macrophages’ phenotype (S2 Fig). No significant impact of NETs on macrophage markers could be observed when compared to their expression on serum-starved cells cultured without NETs. Cells were further loaded with Dil-labeled oxLDL and cultured under normoxic or hypoxic culture conditions for 6 h. Internalized Dil-oxLDL was quantified by flow cytometry (Fig 3A and 3B). For cells cultured under normoxic conditions, oxLDL uptake was additionally confirmed by immunofluorescence (Fig 3C).

In THP-1 macrophages, human NETs significantly increased the number of oxLDL-positive M1- and M2a-like macrophages under both culture conditions. Of note, culture under hypoxia strongly reduced the number of oxLDL-positive M2a-macrophages (Fig 3A).

Similar to THP-1 macrophages, the number of murine M1- and M2a-like cells, taking up oxLDL, was found to increase after stimulation with NETs, although no NETs effects were observed when cells where culture under hypoxia. In M1-like macrophages, hypoxic culture conditions strongly enhanced foam cell formation (Fig 3B). In general, M2a-polarized macrophages cultured under standard conditions were identified to incorporate higher amounts of Dil-oxLDL when compared to M1 macrophages.

**Fig 4:**
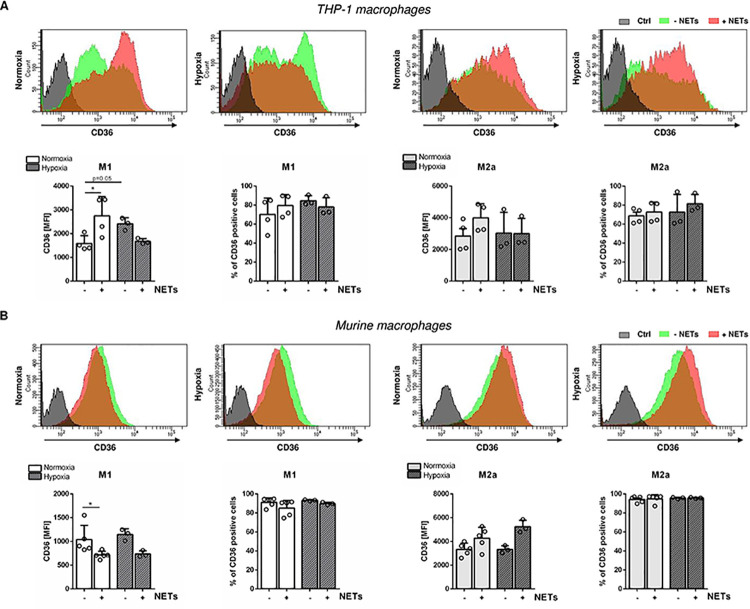
Effect of NETs on CD36 expression on polarized human and murine macrophages. The expression of the oxLDL receptor CD36 on M1- and M2a-like macrophages was quantified by flow cytometry. The median of fluorescence intensity (MFI) as well as the percentage of positive cells were calculated. Representative histograms of 3–5 independent experiments are displayed. *P < 0.05, **P < 0.01, ***P < 0.001 (Mann-Whitney test).

It was previously reported that inhibition of the oxLDL receptor CD36 strongly decreases oxLDL uptake by macrophages [26]. We therefore questioned if increased oxLDL uptake after NETs stimulation is related to upregulated CD36 expression. Cellular CD36 expression was higher in M2a-like macrophages corresponding to increased foam cell formation (Fig 4). Although human NETs did not alter the number of CD36^+^ cells, significant upregulation of CD36 on the surface of M1-like THP-1 macrophages was detected, but not on M2a-like cells (Fig 4A). However, murine NETs significantly decreased the expression of CD36 on M1-like murine macrophages despite increased oxLDL incorporation. Under hypoxia, murine NETs did not significantly changed the expression of CD36 on M1- and M2a-like macrophages (Fig 4B). These data indicate that increased oxLDL uptake after NETs stimulation does not seem to strictly depend on upregulated CD36 expression on macrophages.

### NETs upregulate the expression and secretion of MMP-9 in macrophages

**Fig 5:**
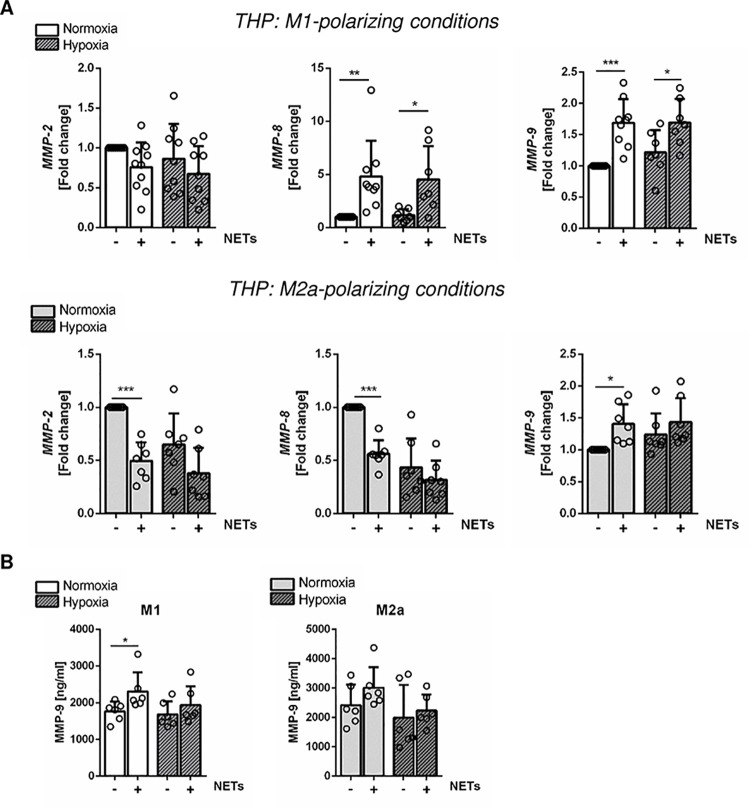
Effect of NETs on the expression of MMPs in polarized human THP-1 macrophages. (A) Relative gene expression of *MMP-2*, *MMP-8* and *MMP-9* in M1—and M2a- polarized human THP-1 macrophages, cultured under normoxia or hypoxia in the presence or absence of human NETs (1000 ng/ml). n = 7–10. (B) MMP-9 protein levels were quantified in culture supernatants by Elisa. n = 6. *P < 0.05, **P < 0.01, ***P < 0.001 (Unpaired *t* test, Mann-Whitney test, One-sample *t* test, Wilcoxon signed-rank test).

The expression of MMPs substantially influences the vulnerability of atherosclerotic plaques. As NETs and macrophages were reported to colocalize in atherosclerotic plaques [27], we next investigated the expression of MMPs in macrophages after exposure to NETs. In THP-M1 cells, we found gene expression of *MMP-8* and *MMP-9* to be strongly increased by NETs, whereas profound inhibition of *MMP-2* and *MMP-8* expression was detected under M2a-polarizing conditions (Fig 5A). However, *MMP-9* expression was increased in NETs-treated THP-M2a macrophages. In turn, hypoxia attenuated NETs effects suggesting that their biological effects strongly depend on oxygen levels. Concomitant with highly upregulated gene expression, THP-M1 cultured under normoxia secreted significantly elevated levels of MMP-9 in the culture supernatant after stimulation with NETs (Fig 5B).

**Fig 6:**
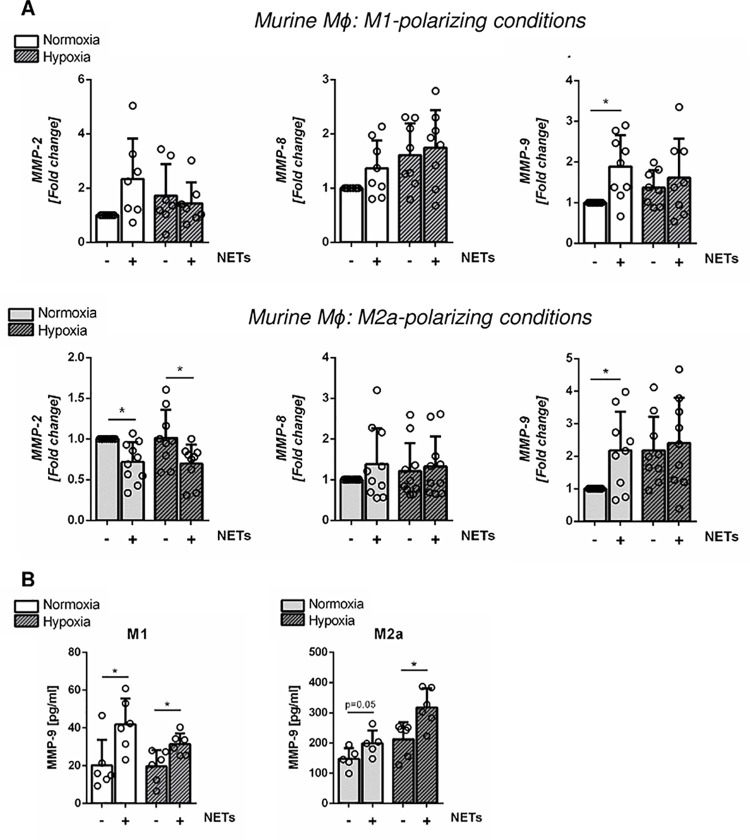
Effect of NETs on the expression of MMPs in polarized murine bone marrow-derived macrophages. (A) Relative gene expression of *MMP-2*, *MMP-8* and *MMP-9* in M1- and M2a-polarized murine macrophages, cultured under normoxia or hypoxia in the presence or absence of NETs (1000 ng/ml) was analyzed by Real-time PCR. n = 7–10. (B) MMP-9 protein levels were quantified in culture supernatants by Elisa. n = 6. *P < 0.05 (One-sample *t* test, Mann-Whitney test).

Murine M1-like macrophages exposed to murine NETs under normoxia showed upregulated *MMP-9* expression, whereas M2a-like macrophages displayed reduced *MMP-2* and elevated *MMP-9* expression (Fig 6A). However, NETs stimulation resulted in increased MMP-9 secretion from macrophages, regardless of the polarization state of the cells and oxygen levels (Fig 6B).

## Discussion

Macrophages represent key players in the genesis and progression of atherosclerosis in mice and humans. It has previously been reported that macrophages exhibit an M2 phenotype at early disease stages and become M1 during plaque progression [28]. The M2 phenotype was associated with the resolution of plaque inflammation and regression of atherosclerosis [8, 9]. During the past decade, many studies reported the presence of NETs in human and murine atherosclerotic lesions [19, 20, 27], but their biological functions remain incompletely understood.

In the present *in vitro* study, we have compared the impact of NETs on human and murine macrophages in respect to the expression of cytokines and MMPs as well as foam cell formation. One important finding of our study was the opposite effect of NETs on the expression of pro-inflammatory cytokines by human and murine macrophages. NETs enhanced the expression of pro-inflammatory genes in THP-1 macrophages cultured under M1-polarizing conditions and standard oxygen levels but not in murine M1-like cells. On the contrary, the expression of *IL-1β*, *TNF-α* and *IL-6* was significantly suppressed in murine M1-like cells under hypoxia largely supporting the results of our previous report [21]. Gene expression levels of IL-1β visibly correlated with protein secretion. These findings contradict the results of an earlier study showing an increase of pro-inflammatory IL-1α, IL-1β and IL-6 in atherosclerotic lesions of mice containing NETs [19]. Similarly, Josefs *et al*. recently reported that NETs-positive areas in atherosclerotic plaques of mice express higher levels of the M1 marker gene *iNOS* suggesting that NETs promote an M1-like phenotype [27]. However, the presence of NETs in atherosclerotic lesions does not provide supporting evidence for their pro-inflammatory actions. The effect of NETs was shown to become reversed by the treatment of mice with *DNase I* [27] or peptidylarginine (PAD) inhibitors [29, 30]. It is noteworthy to consider that *DNase I* does not specifically degrade NETs but rather cell-free DNA (cfDNA) released by necrotic cells, which in turn acts highly pro-inflammatory [21, 31]. Additionally, a wide range of side effects of commonly used PAD inhibitors in mice, like inhibition of T cell proliferation and suppression of dendritic cells and smooth muscle cells activation needs to be considered for the interpretation of findings obtained with PAD inhibitors [32–34]. Of note and in accordance with prior work [35], our data presented here also demonstrate that human NETs significantly enhance pro-inflammatory cytokine expression in human THP-M1 macrophages.

Studies have indicated that foam cells play a major role in plaque formation during atherosclerosis and promote disease progression [36, 37]. Here, we show for the first time that NETs strongly increase the number of macrophages that take up oxLDL independently on macrophages’ phenotype. In addition, previous studies reported NETs formation after stimulation of human [38, 39] neutrophils with oxLDL. As neutrophils and monocytes / macrophages come in close cell-to-cell contact within the atherosclerotic lesions [27, 40], NETs derived from e.g. oxLDL-stimulated neutrophils may probably contribute to oxLDL uptake by macrophages and foam cell formation in both mice and humans. In line with the findings of a previous report [41], the number of oxLDL-positive M2a-like macrophages was found to be higher compared to M1-like cells showing positive correlation with increased CD36 expression on these cells. In contrast, NETs-mediated foam cell formation does not seem to depend on surface CD36 expression. As NETs diminished the expression of pro-inflammatory cytokines in M1-like murine macrophages, it is likely that NETs-triggered transdifferentiation from M1 cells to M2 cells [21] may, at least in mice, contribute to lipid accumulation. However, we did not investigate the expression of additional oxLDL receptors or determined the impact of NETs on cholesterol efflux, representing a limitation of this study. Besides, in human THP-1 cells, different mechanisms could be involved and it is still unclear which molecules regulate NETs-mediated foam cell formation. In this respect, a role of NETs-associated citrullinated histones has recently been suggested. The authors demonstrated that citrullinated histones accelerate LDL aggregation and foam cell formation in dependence of the citrulline content [42]. As PMA, which has been used in this study to stimulate neutrophils, was found to enhance histone citrullination [43, 44] too, it is likely that increased oxLDL incorporation is mediated by NETs-associated citrullinated histones.

Atherosclerotic lesions represent a hypoxic milieu that favor the uptake of oxLDL by macrophages [25]. Therefore, in human atherosclerotic plaques, the hypoxic regions are rich in macrophages and foam cells [45]. Here, hypoxia did not change oxLDL uptake by human THP-1 macrophages and M2a-like murine cells, but highly increased oxLDL incorporation by M1-polarized murine macrophages. Additionally, the effect of NETs on foam cell formation also depended on the hypoxic environment, whereby lower oxygen levels suppressed NETs-mediated oxLDL uptake by murine macrophages.

MMPs produced by macrophages contribute to plaque rupture, atherothrombosis and MI [46]. Here, NETs increased the expression and secretion of MMP-9 in both human and murine macrophages, independently on their phenotype. MMP-9 is involved in all stages of atherosclerosis and MMP-9 deletion is associated with reduced plaque size, macrophage content and collagen deposition in aortic lesions of *ApoE*^*-/-*^ mice [47]. In humans, plasma MMP-9 levels were reported to be higher in patients with ruptured plaque compared with patients without ruptured plaques. Therefore, MMP-9 was suggested to represent an independent predictor of plaque rupture [48–50]. On the other hand, NETs were found to diminish MMP-2 gene expression in human and murine macrophages under M2a-polarizing culture conditions. It is known that MMP-2 plays a role in promoting atherosclerosis, since atherogenesis is reduced in MMP-2-deficient *ApoE*^*−/−*^ mice [51]. Thus, by regulating the expression of MMPs, it may be assumed that NETs exert protective or harmful effects depending on the context.

The results of this study reflect the discrepancy between human and murine macrophages, which has already been characterized in detail. Moreover, the use of THP-1 monocytes instead of peripheral blood-derived monocytes represents an important limitation of our study. Although previous research has shown that human peripheral blood-derived macrophages and THP-1 macrophages were more closely related to each other than to mouse macrophages, some discrepancies between these cells have also been observed [52]. In this respect, by comparing human THP-1 macrophages and human monocytes-derived macrophages some investigators concluded that THP-1 macrophages represent an appropriate model to study the M1 polarization but less the M2 polarization [53, 54]. For this reason, additional studies should be conducted in the future to prove the effect of NETs on primary human macrophages or iPS cells-derived macrophages, which were found to share many characteristics with primary human cells and display less heterogeneity in comparison to primary macrophages [52].

Overall, our results presented herein suggest that the effects of NETs on macrophages are manifold, depending on the origin of the cells, polarization state and oxygen level. NETs increase the expression of pro-inflammmatory cytokines in human THP-M1 macrophages but strongly suppress their expression in murine bone marrow-derived M1 macrophages. Additionally, we provide evidence for NETs-mediated increased oxLDL uptake by M1 and M2 macrophages *in vitro*. However, hypoxia may dampen or alter the effects of NETs on macrophages to some extent. Taking into account that NETs enhanced MMP-9 expression and release from macrophages *in vitro*, we propose that NETs play an important role in plaque stability, vulnerability and progression. Our findings may provide new insights on NETs-mediated atherogenesis and disease progression.

## Supporting information

S1 Fig Characterization of THP-1- derived M1- and M2a-polarized macrophages. (A) Surface expression of CD11b on PMA-treated, differentiated macrophages (M0) quantified by flow cytometry. n = 4. (B) The surface expression of M1 (CD197) and M2 (CD163, CD206) marker proteins on polarized THP-1 macrophages was quantified by flow cytometry. One representative histogram overlay of four independent experiments is depicted. (C) Relative gene expression of *IL-1β*, *TNF-α*, *IL-6* and *IL-12* in polarized macrophages quantified by Real-time PCR. n = 5. *P < 0.05, **P < 0.01 (Mann-Whitney test).
(TIF)

S2 Fig Impact of serum-free culture conditions and NETs on the expression of M1- and M2-specific markers on polarized macrophages. M1- and M2a-polarized human (A) and murine (B) macrophages were cultured under standard culture conditions in serum-free medium for 24 h with or without NETs (1000 ng/ml). The expression of CD197 (M1), CD163 and CD206 (M2) were analyzed by flow cytometry. n = 3. *P < 0.05 (Kruskal-Wallis test with Dunn’s post-hoc test).
(TIF)
