# Supplementary figures and images for: Neutrophil extracellular traps modulate inflammatory markers and uptake of oxidized LDL by human and murine macrophages
Source: PLoS One. 2021 Nov 19;16(11):e0259894. doi: 10.1371/journal.pone.0259894 (PMC8604363; doi:10.1371/journal.pone.0259894)

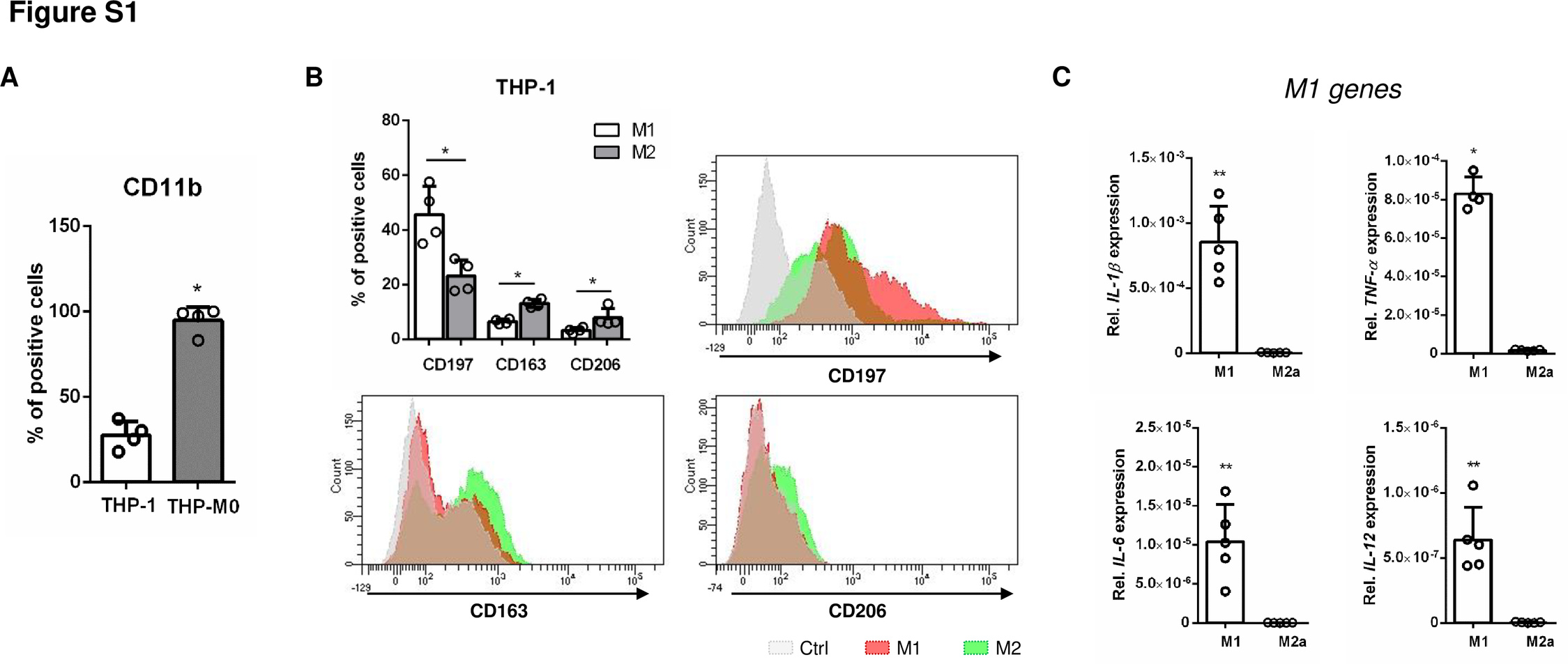

Supplement: S1 Fig — (A) Surface expression of CD11b on PMA-treated, differentiated macrophages (M0) quantified by flow cytometry. n = 4. (B) The surface expression of M1 (CD197) and M2 (CD163, CD206) marker proteins on polarized THP-1 macrophages was quantified by flow cytometry. One representative histogram overlay of four independent experiments is depicted. (C) Relative gene expression of IL-1β, TNF-α, IL-6 and IL-12 in polarized macrophages quantified by Real-time PCR. n = 5. *P < 0.05, **P < 0.01 (Mann-Whitney test). (TIF) [file pone.0259894.s001.tif]

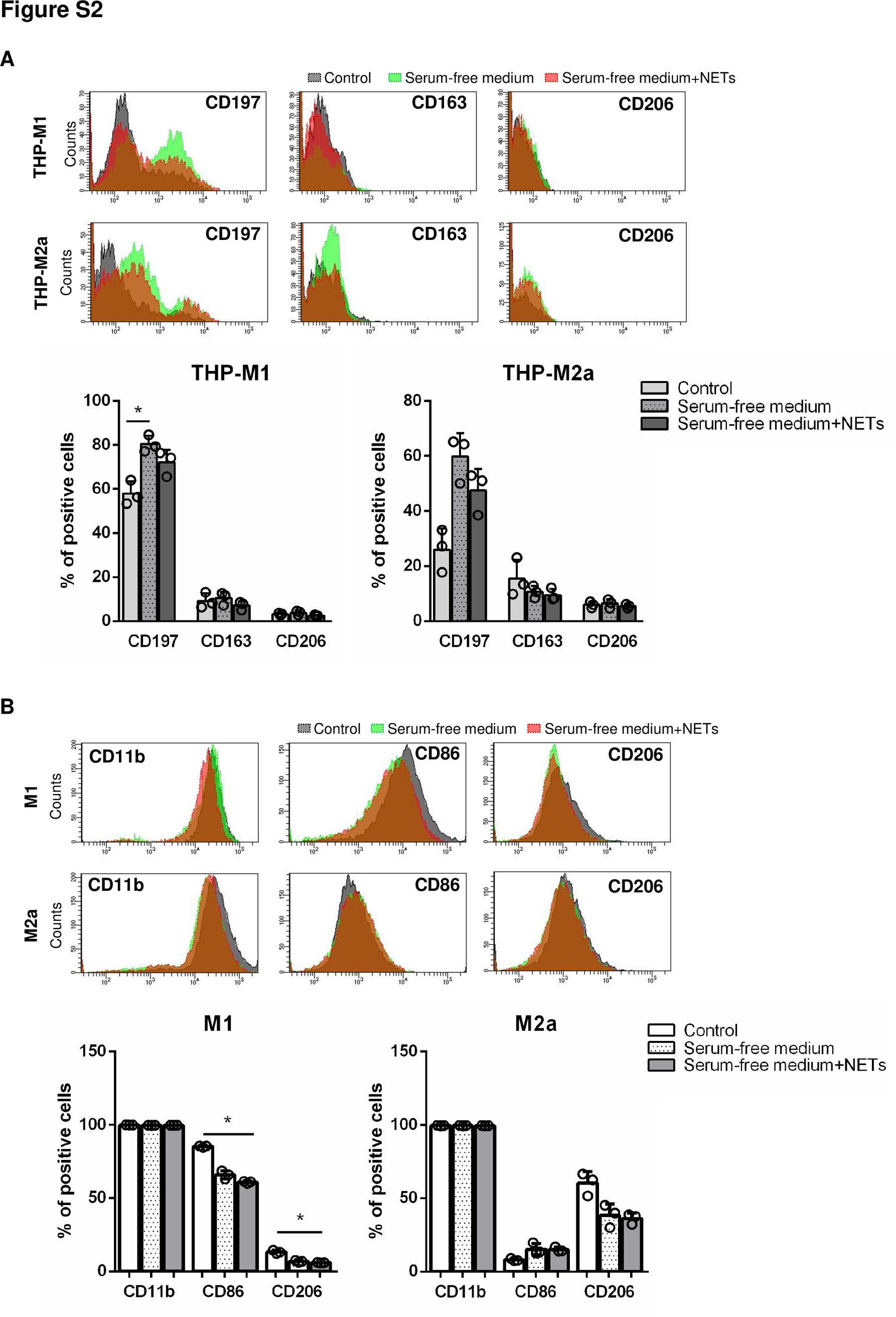

Supplement: S2 Fig — M1- and M2a-polarized human (A) and murine (B) macrophages were cultured under standard culture conditions in serum-free medium for 24 h with or without NETs (1000 ng/ml). The expression of CD197 (M1), CD163 and CD206 (M2) were analyzed by flow cytometry. n = 3. *P < 0.05 (Kruskal-Wallis test with Dunn’s post-hoc test). (TIF) [file pone.0259894.s002.tif]
